# Development and optimization of electrophoretically deposited octacalcium phosphate–collagen film as bone analogues

**DOI:** 10.1093/rb/rbaf136

**Published:** 2026-02-06

**Authors:** Katrina J Staunton-Mann, Gengyao Wei, Thomas Kress, David J Barrett, Melinda J Duer, Ruth E Cameron, Serena M Best

**Affiliations:** Department of Materials Science & Metallurgy, University of Cambridge, Cambridge CB3 0FS, UK; Department of Materials Science & Metallurgy, University of Cambridge, Cambridge CB3 0FS, UK; Yusuf Hamied Department of Chemistry, University of Cambridge, Cambridge CB2 1EW, UK; Department of Materials Science & Metallurgy, University of Cambridge, Cambridge CB3 0FS, UK; Yusuf Hamied Department of Chemistry, University of Cambridge, Cambridge CB2 1EW, UK; Department of Materials Science & Metallurgy, University of Cambridge, Cambridge CB3 0FS, UK; Department of Materials Science & Metallurgy, University of Cambridge, Cambridge CB3 0FS, UK

**Keywords:** collagen, mineralization, bone model, electrophoretic deposition

## Abstract

The hierarchical architecture of bone, characterized at the nanoscale, is defined by mineralized collagen fibrils with a disordered, hydrated, carboxylate-rich mineral surface, presenting a complexity often absent in conventional hydroxyapatite (HA) models. Here, we report the design of biomimetic octacalcium phosphate (OCP)–collagen films that reproduce both the crystalline mineral core and the citrate-rich disordered surface of native bone. Phase-pure OCP and citrate-incorporated OCP (OCP-CIT) were synthesized via pH-regulated hydrolysis of α-tricalcium phosphate (α-TCP) under physiological conditions, with citrate inducing structural heterogeneity analogous to natural bone mineral. Electrophoretic deposition facilitated the integration of these minerals into collagen films, aided by hyaluronic acid (HyAc), which stabilized colloidal suspensions by adjusting the zeta potential from −5 to −25 mV and dialysis against DI water by lowering conductivity from ∼30 to 0.01 mS/cm. The resulting films exhibited a collagen–mineral composite composed predominantly of apatitic orthophosphates (74% by ³^1^P nuclear magnetic resonance (NMR) spectroscopy), with citrate-directed mineral nucleation and HyAc-promoted collagen mineralization. Solid-state NMR rotational-echo double resonance (REDOR) experiments highlighted the essential function of HyAc in establishing proximity between mineral and collagen, absent in citrate-only systems, thereby emulating bone’s interfacial organization. The work establishes a scalable film-based strategy for creating physiologically relevant bone analogues, with implications for advancing bone graft materials and disease models.

## Graphical abstract

**Figure:**
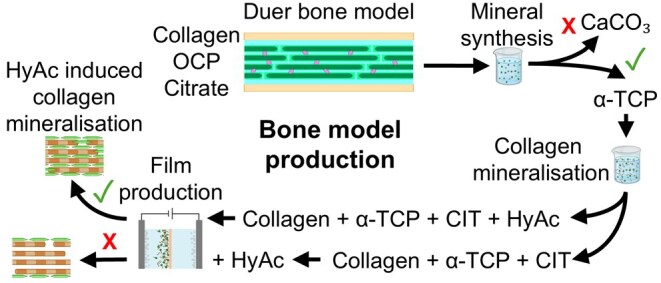

## Introduction 

Bone is a complex hierarchical composite material with remarkable mechanical properties that arise from the intricate organization of its organic and inorganic components. At the nanoscale, bone consists of mineralized collagen fibrils in which nanoscopic calcium phosphate platelets are embedded both within and around the fibrils, forming an intimately integrated collagen–mineral interface [1]. This interface plays a crucial role in determining bone’s macroscopic mechanical properties, governing load transfer, toughness and resistance to fracture [2, 3]. Despite extensive research in biomaterials and bone tissue engineering, replicating the structure, composition and functionality of native bone *in vitro* remains a significant challenge. The development of accurate bone models would not only enhance our fundamental understanding of bone mineralization but also improve the design of bone graft materials and enable more physiologically relevant platforms for studying bone diseases.

Historically, hydroxyapatite (HA) has been the primary focus of bone biomimetics, given its chemical similarity to the mineral phase of bone. However, HA-based models fail to capture the full complexity of bone mineralization, particularly the carboxylate-rich, hydrated, disordered surface layer observed in natural bone. Recent advances in solid-state nuclear magnetic resonance (NMR) and electron microscopy have revealed that native bone mineral platelets possess an apatitic core surrounded by a disordered, hydrated, carboxylate-rich surface [4–12]. Citrate molecules, in particular, have been identified as key regulators of bone mineral properties, restricting the growth of calcium phosphate crystals and stabilizing the hydrated layer [5, 7, 13]. This understanding has led to the Duer bone mineral model [14], which proposes that bone mineral consists of alternating layers of ordered apatite and hydrated disordered regions enriched with citrate. To develop accurate *in vitro* bone models, it is therefore essential to replicate not only the apatitic core but also this hydrated, carboxylate-rich surface environment, a feature absent in conventional HA-based systems.

One promising approach to reproducing this structural complexity involves the use of octacalcium phosphate (OCP) and its carboxylate-substituted derivatives, such as citrate-incorporated OCP (OCP-CIT) [4]. OCP exhibits a layered structure with alternating apatitic and hydrated regions, making it an excellent analogue for the bone mineral model [15]. Moreover, OCP is a known precursor to HA, capable of undergoing epitaxial transformation under physiological conditions [16, 17]. However, some existing OCP synthesis methods are conducted at elevated temperatures [18], which are incompatible with the presence of collagen, as collagen denatures above 37°C. To enable the mineralization of collagen suspensions and the development of biomimetic bone models, OCP synthesis must therefore be adapted to physiological conditions.

Two synthesis routes have been explored in the literature for producing OCP and OCP-carboxylates: the reaction of calcium carbonate (CaCO_3_) with phosphoric acid [19] and α-tricalcium phosphate (α-TCP) [15, 20]. The presence of carbonate ions during OCP synthesis offered by the CaCO_3_ method may provide a route for carbonate substitutions, another key component of bone mineral. This method has primarily been conducted at 60°C, and lowering the synthesis temperature to 37°C can slow reaction kinetics and lead to the formation of undesired phases, such as dicalcium phosphate dihydrate (DCPD) and HA, unless pH is carefully controlled [21–23]. Conversely, the α-TCP hydrolysis method has been successfully implemented at 37°C [15, 20], but optimal pH conditions must be identified to ensure high phase purity and avoid the formation of unwanted byproducts such as calcium citrate.

Beyond synthesizing biomimetic minerals, a second critical challenge is integrating these minerals into collagen matrices to replicate the hierarchical structure of native bone. One promising approach to achieving this is electrophoretic deposition (EPD), a materials processing technique that enables the formation of dense, free-standing collagen films [24]. Unlike traditional vacuum-based methods, which risk collapsing the hydrated layers of OCP-CIT [4], EPD preserves the hydrated mineral surface, making it particularly well-suited for bone biomimetics [25]. However, successful EPD requires careful modulation of the mineral–collagen suspension properties, including zeta potential and conductivity, to ensure homogeneous deposition and prevent electrolysis-induced defects.

Hyaluronic acid (HyAc) emerges as a potential processing agent in this context. HyAc is a negatively charged glycosaminoglycan (GAG) present in the bone extracellular matrix (ECM), known to modulate mineralization [26], promote osteogenesis [27–29] and enhance cell response to biomaterials [27]. Previous studies have demonstrated that HyAc interacts with collagen fibrils through hydrogen bonding, altering their zeta potential and facilitating mineral nucleation and deposition [30, 31]. In collagen mineralization studies, polyanionic molecules such as poly-aspartic acid have been widely used to guide mineral infiltration into collagen fibrils, but the role of HyAc as a direct processing agent remains less explored. This study investigates whether HyAc can modulate the stability and charge of OCP-based collagen suspensions, thereby improving EPD efficiency and enhancing the formation of a biomimetic collagen–mineral interface.

This work aims to establish a unified approach that bridges fundamental mineral synthesis with applied biomaterial processing to develop an advanced bone-mimetic model. The study is structured around two key objectives: (1) Optimizing the synthesis routes of biomimetic OCP-based minerals under physiological conditions via pH control and synthesis time; (2) developing mineralized collagen films using EPD and establishing strategies to modulate the conductivity and zeta potential of mineralized collagen suspensions. We employ a suite of characterization techniques to verify phase purity, crystallinity and the successful incorporation of citrate in OCP. Notably, advanced NMR techniques allow us to shed light on the organization of the collagen–mineral–citrate interface of mineralized collagen films. By integrating biomimetic mineral synthesis with advanced materials processing, this work bridges the gap between fundamental chemistry and applied biomaterials research. The findings will advance bone grafting strategies, facilitate the development of bone disease models and contribute to the broader understanding of bone mineralization mechanisms.

## Materials and methods

### Reaction of CaCO_3_ with phosphoric acid

For the synthesis of OCP, CaCO_3_ (1.604 g) and orthophosphoric acid (0.6 mL) were added to deionized water (200 mL) with constant stirring at 500 rpm and maintained at 37°C in a water bath. The pH of the suspension was maintained at 6.5 by dropwise addition of 1 M hydrochloric acid. To synthesize OCP-CIT, citric acid (3.84 g) was dissolved in deionized water (200 mL) and the pH was adjusted to 7 using sodium hydroxide, prior to the addition of CaCO_3_ (1.604 g) and orthophosphoric acid (0.6 mL) to achieve a calcium-to-citrate concentration ratio of 1:1.25. After various synthesis times, the reaction product was gravity-filtered, washed with deionized water and air-dried.

### Hydrolysis of α-TCP

For the synthesis of OCP, α-TCP (0.8 g, 8 g/L, Geistlich) was added to deionized water (100 mL) while stirring at 200 rpm and maintained at 37°C in a water bath for 24 h. For the synthesis of OCT-CIT, citric acid (2.643 g) was added to deionized water (55 mL) while stirring at 200 rpm and maintained at 37°C in a water bath, and the pH was adjusted to 5.52 using sodium hydroxide. α-TCP (0.836 g) was then added, and the suspension was left to stir for 48 h. Both reactions were conducted under two pH conditions: (1) initial pH adjustment to 6.50 and then uncontrolled; (2) a constant pH at 6.50 for the synthesis duration by dropwise addition of 1 M hydrochloric acid. The reaction product was gravity-filtered, washed with deionised water and air-dried.

### Mineralizing collagen suspension

Two types of mineralized collagen film were produced, OCP-CIT/COLL and OCP-CIT/COLL/HyAc. The materials included within the mineralization suspension and steps taken for the preparation of EPD are outlined in Table 1 and detailed in subsequent sections.

**Table 1:** The materials within the mineralization mixture and the EPD preparation steps taken for each mineralized collagen film

Sample name	Materials in mineralization suspension				EPD preparation		
	α-TCP	Collagen	Citrate	HyAc	Dialysis	HyAc	Collagen
OCP-CIT/COLL	✓	✓	✓	x	✓	✓	✓
OCP-CIT/COLL/HyAc	✓	✓	✓	✓	✓	x	✓

#### Collagen suspension preparation

Collagen suspension (0.5 or 1 wt.%) was prepared by hydrating insoluble bovine dermal collagen (Devro) in 0.05 M acetic acid for 72 h at 4°C followed by homogenization using a blender. The homogenized slurry was dialyzed against deionized water for 48 h with 8 water changes and a suspension-to-water ratio of 1:20.

#### Collagen suspension mineralized with OCT-CIT

Citric acid (1.897 g) was added to collagen suspension (1 wt.%, 60 mL) under constant stirring at 37°C, and the pH was adjusted to 5.5 using sodium hydroxide. After stirring for 1 h, α-TCP powder (0.6 g, 10 g/L) was added to the collagen suspension and pH adjusted to 6.50 before stirring for 4 days. Following mineralization, the suspension was homogenized on ice (30 s). To adjust the conductivity and zeta potential of the suspension, HyAc sodium salt (streptococcus equi, 91%, Alfa Aesar, USA) was added to the suspension whilst stirring at 200 rpm for 1 h, reaching final concentrations of 0.25–1 mg/mL. It is worth noting that HyAc was only used as a processing agent for the subsequent EPD and not incorporated in the collagen–mineral interface due to the low temperature. The suspension was dialyzed against deionized water, causing swelling to 90 mL. About 0.5 wt.% collagen suspension (30 mL) was added to the dialyzed mineralized collagen, homogenized on ice (90 s) and centrifuged to allow the removal of excess water (30 mL), followed by homogenization on ice (30 s).

#### Collagen suspension mineralized with OCT-CIT and HyAc

Citric acid (1.897 g) was added to collagen suspension (1 wt.%, 60 mL) under constant stirring at 37°C, and the pH was adjusted to 5.5 using sodium hydroxide. After stirring for 1 h, HyAc sodium salt (0.03 g, 0.5 mg/mL) was added to the suspension to be incorporated in the collagen–mineral interface. After stirring for 1 h, α-TCP powder (0.6 g, 10 g/L) was added to the collagen suspension and pH adjusted to 6.50 before stirring for 4 days. Following mineralization, the suspension was homogenized on ice (30 s) and dialysed against deionized water. 0.5 wt.% collagen suspension (20 mL) was added to the dialysed mineralized collagen suspension whilst stirring and homogenized on ice (90 s).

### EPD of mineralized collagen films

**Figure 1:**
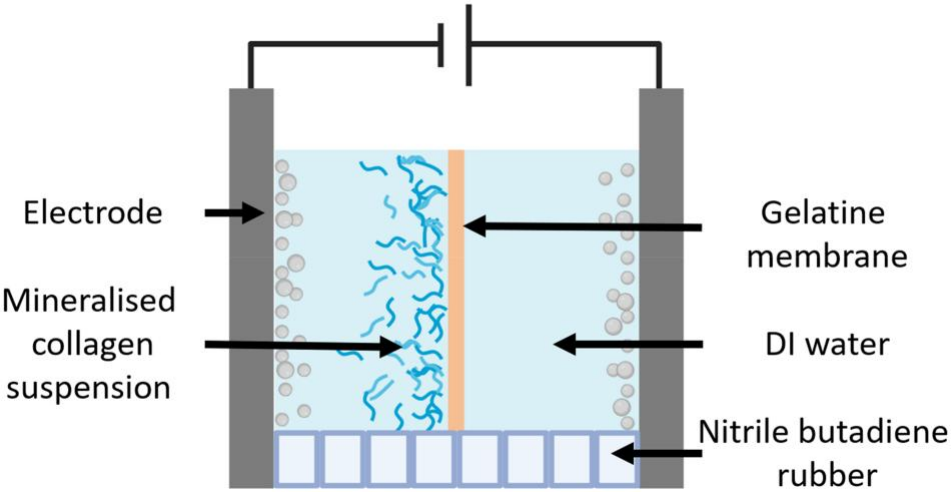
Schematic of the EPD cell containing a suspended gelatine membrane separating the collagen suspension (cathode) and deionized water (anode).

EPD was carried out in a custom-built EPD cell, as depicted in Figure 1, with a distance of 1.2 cm between 316 L stainless steel electrodes. A gelatine membrane (thickness = 85 µm) was suspended between nitrile butadiene rubber spacers at the centre of the EPD cell. The membrane was produced by air drying a 5 wt.% gelatine solution in a silicone mould. The gelatine solution was dialysed against deionized water before drying. A B2901A Source Meter Unit (Keysight Technologies) was connected to the electrodes as the voltage source. Equal volumes of mineralized collagen suspension and deionized water were pipetted into each compartment of the EPD cell. A voltage of 10 V was applied for 10 min after which the liquids were replenished, and the deposition was continued for another 10 min. Subsequently, the EPD cell was gently deconstructed and the collagen films deposited on the gelatine membrane were submerged in deionized water at 37°C for 1 h to remove the gelatine membrane. The released collagen films were gently rinsed with deionized water and air-dried.

### X-ray diffraction

The minerals were ground in a pestle and mortar and mineralized collagen films were secured to sample holders using petroleum jelly for X-ray diffraction (XRD, Bruker D8 DAVINCI). XRD was carried out at an operating voltage of 40 kV, an X-ray wavelength of α1 = 1.54059744 Å, a current of 40 mA, a step size of 0.02° from 2*θ* = 3°–60° and a sample rotation speed of 30 rpm. Data were collected at constant sample length and the over-illumination error was corrected in DIFFRAC.EVA (Bruker) software. HighScore Plus (Malvern Panalytical, UK) was used to identify peaks in the XRD traces which were matched with the Inorganic Crystal Structure Database (ICSD) with reference patterns noted where appropriate. Rietveld refinement was applied to assess the phase purity of the samples.

### Nuclear magnetic resonance

Solid-state NMR measurements were performed on a Bruker 400 MHz proton frequency (9.4 T) Avance spectrometer equipped with a standard double or triple resonance probe at frequencies of 400.3 MHz (^1^H), 162.1 MHz (^31^P) and 100.6 MHz (^13^C). Samples were packed in 4 mm zirconia rotors and rotated at a magic angle spinning (MAS) rate of 10 kHz at room temperature. The ^13^C measurements were referenced to methylene glycine signal at 43.1 ppm relative to tetramethylsilane at 0 ppm and ^31^P measurements were referenced to alendronate at 17.7 ppm, which corresponds to crystalline HA at 2.8 ppm relative to 85% phosphoric acid at 0 ppm. SPINAL64 decoupling was used during signal acquisition.

^31^P high power decoupling (HPDEC) was carried out at a ^31^P 90° pulse length of 3.5 µs with a 15 s recycle time.^31^P cross polarization (CP) was carried out at a ^1^H 90° pulse length of 2.5 µs, and the ^1^H–^31^P CP contact time was 2 ms with 5 s recycle delay.^13^C{^31^P} Rotational-Echo Double Resonance (REDOR) was carried out with 180° pulses of length 6.5 µs, 50 µs period and dephasing time of 2.9 ms for OCP-CIT samples and 9.9 ms for mineralized collagen samples. The power of the ^31^P pulses was set to zero in the reference spectrum.

### Zeta potential and conductivity

Zeta potential and conductivity measurements were carried out using laser Doppler electrophoresis with a Zetasizer Nano-ZS (Malvern Instruments) at 25°C. Unless stated otherwise, values quoted were the average and standard deviation of 3 data sets for each sample. Conductivity measurements were undertaken using a Zetasizer Nano-ZS, with an accuracy of ±10%, which is higher than the standard deviation of the samples. Therefore, the errors were calculated using the manufacturer’s recommendations of ±10%.

## Results

### Synthesis of OCP and OCP-CIT minerals

The CaCO_3_ and α-TCP reaction methods were optimized to maximize phase purity of OCP and OCP-CIT before establishing the optimal method to produce these minerals at 37°C.

#### CaCO_3_ route

The synthesis of OCP at 37°C after 5 h resulted in the formation of DCPD when leaving the pH uncontrolled, as shown in Figure 2A. At this physiologically relevant temperature, DCPD formation was avoided by maintaining the pH at 6.50 for the duration of the synthesis. To improve the proportion of OCP in the samples, the synthesis time was increased from 5 h (Figure 2B, OCP % = 79.8 ± 2.8, *N* = 2) to 24 h (Figure 2C, OCP % = 89.6 ± 10.0, *N* = 2). Beyond this synthesis time, there were stronger reflections in the apatitic regions, suggesting potential transformation into HA (Figure 2D). To assess the crystallinity of OCP formation, the full-width half maximum (FWHM) of the OCP (1 0 0) reflection was measured, as shown in Table 2. This demonstrated a decrease in FWHM with increasing synthesis time, indicating an increase in crystallinity.

**Figure 2:**
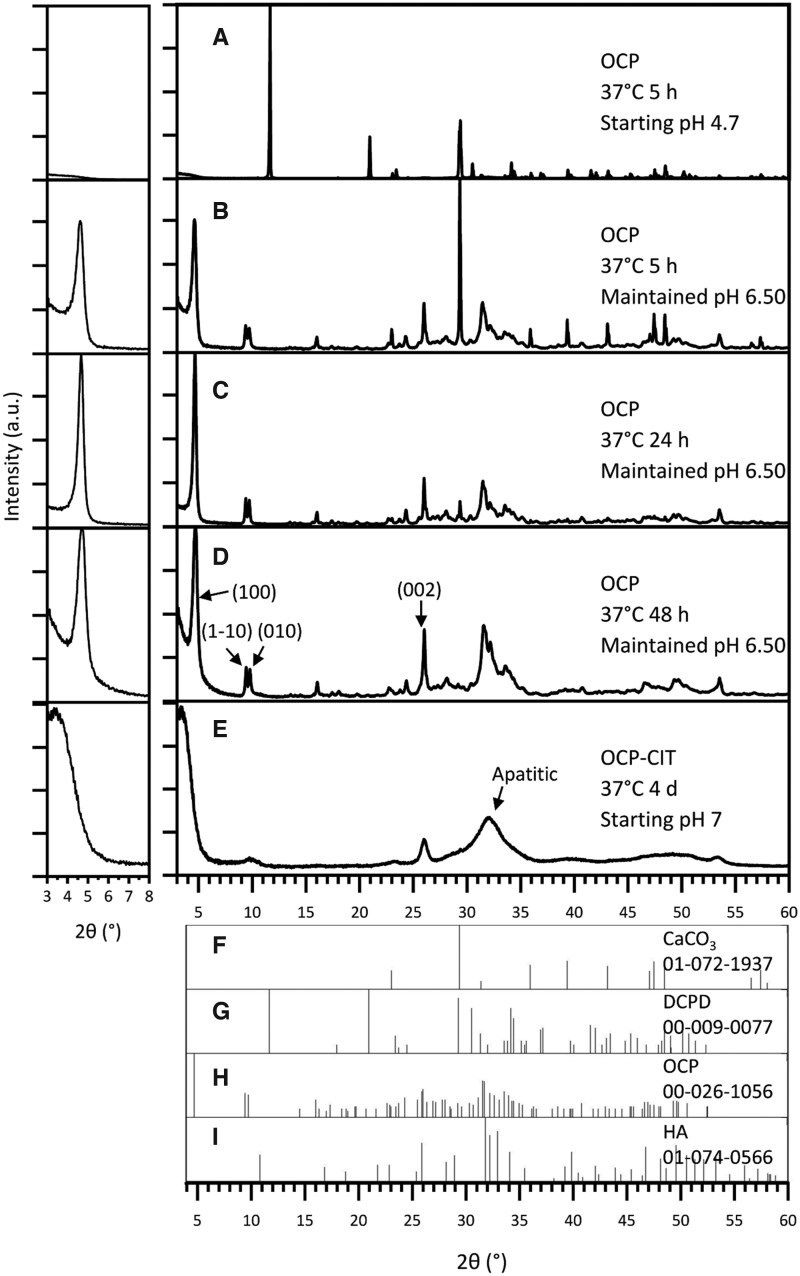
The XRD pattern of products from OCP (CaCO_3_ route) synthesis at 37°C, with noted pH conditions and synthesis time. With no pH adjustments, the synthesis temperature of 37°C resulted in DCPD formation (**A**). Maintaining the pH at 6.50 during synthesis resulted in OCP formation at 37°C, but the synthesis time required increased from 5 h (**B**) to 24 h (**C**) to ensure reaction completion. Beyond 24 h, HA contamination was observed (**D**). OCP-CIT formation was only observed after 4 days of hydrolysis, showing broad peaks (**E**). Due to OCP’s characteristic large (1 0 0) peak around 2*θ* = 4°, the pattern is shown on a magnified scale in the inset (left) for clarity. Reference patterns for CaCO_3_, DCPD, OCP and HA are shown in (**F**–**I**), respectively.

**Figure 2:**
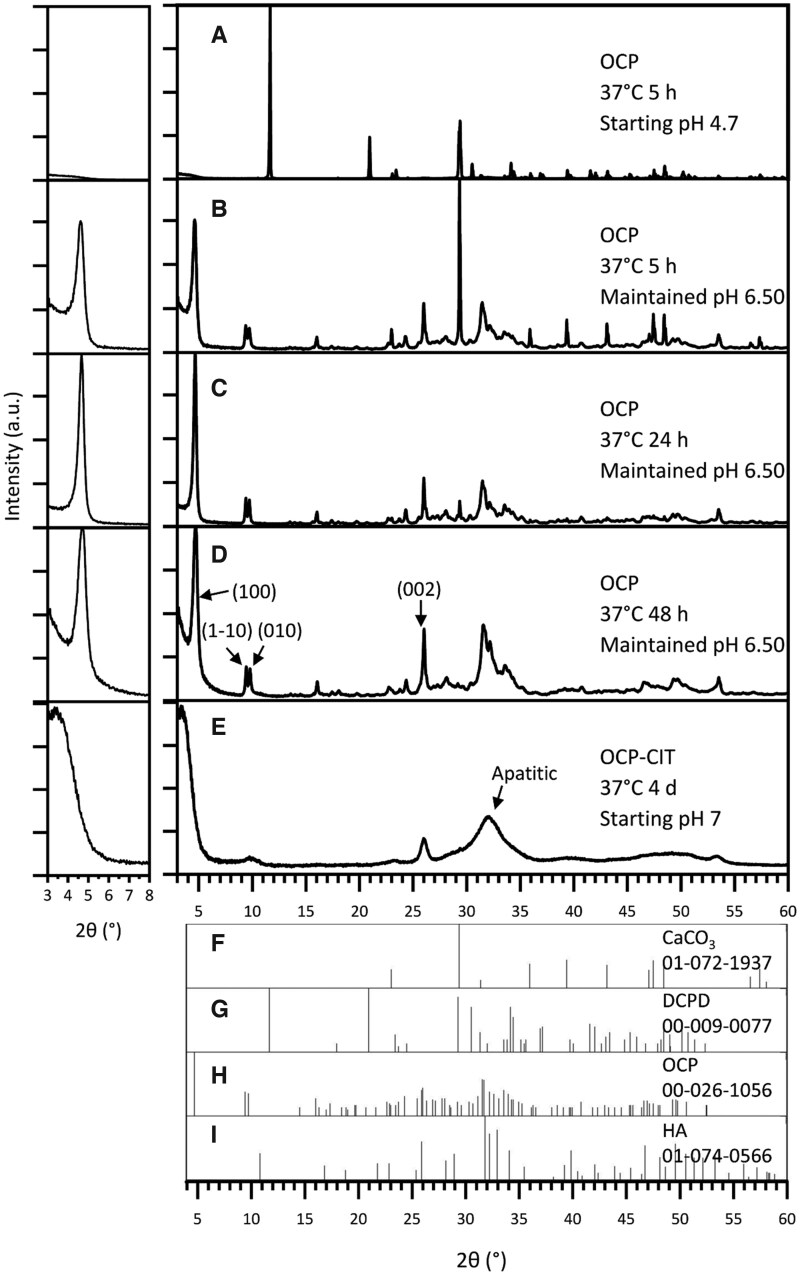
Continued.

**Figure 2:**
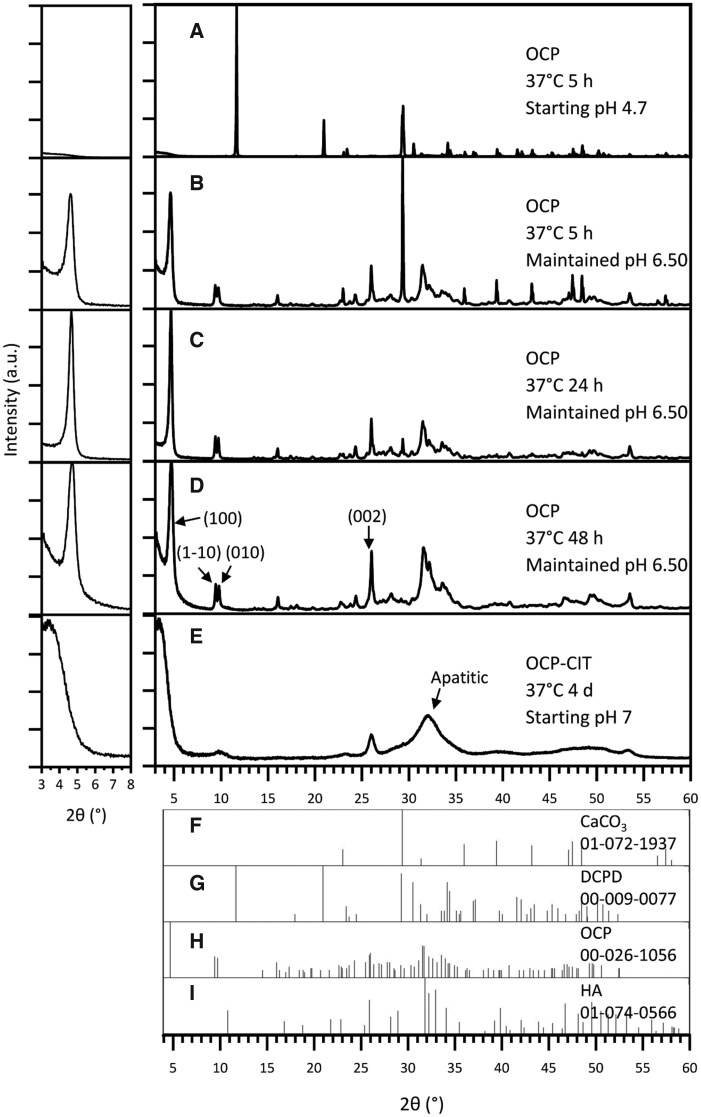
Continued.

**Figure 2:**
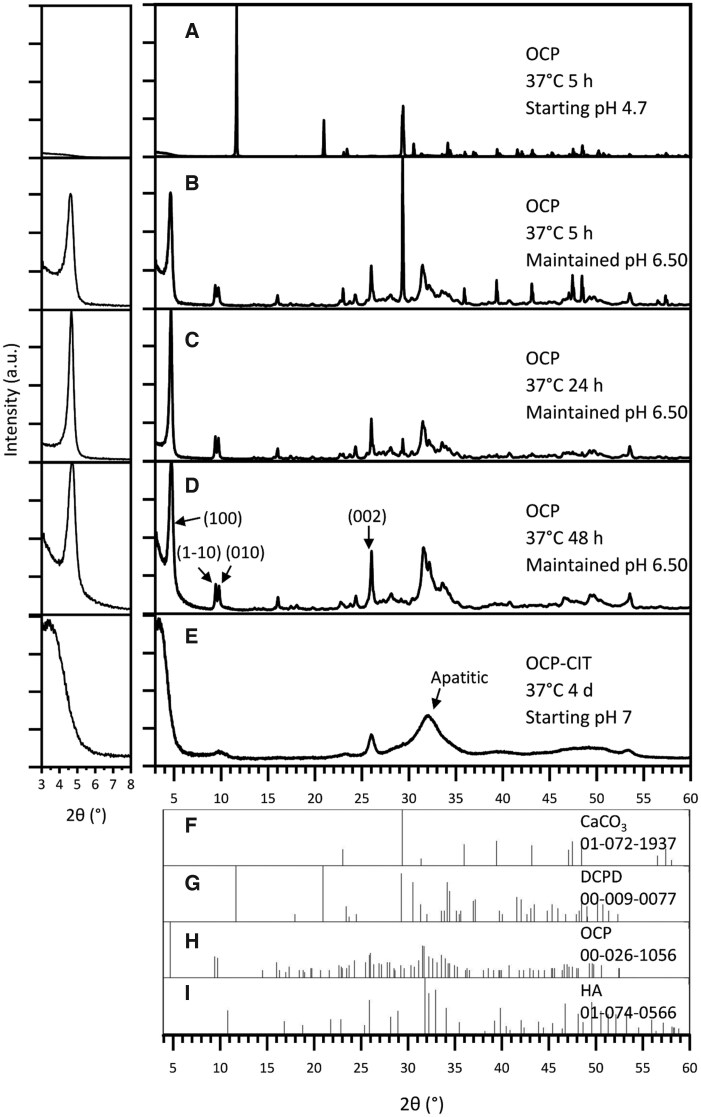
Continued.

**Figure 2:**
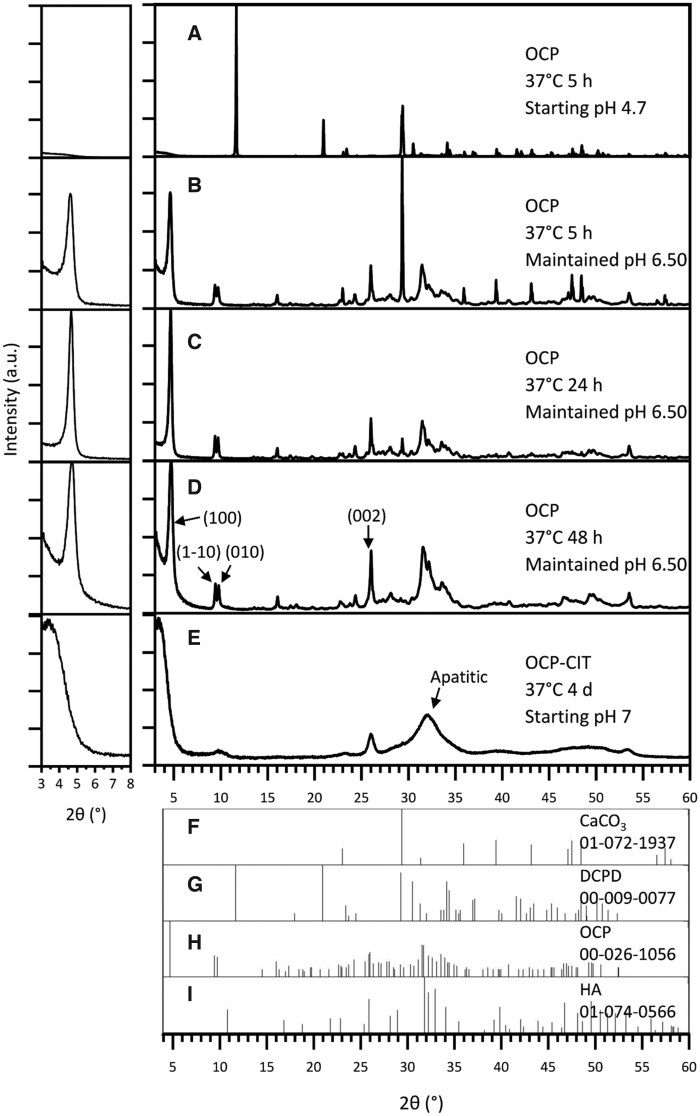
Continued.

**Figure 2:**
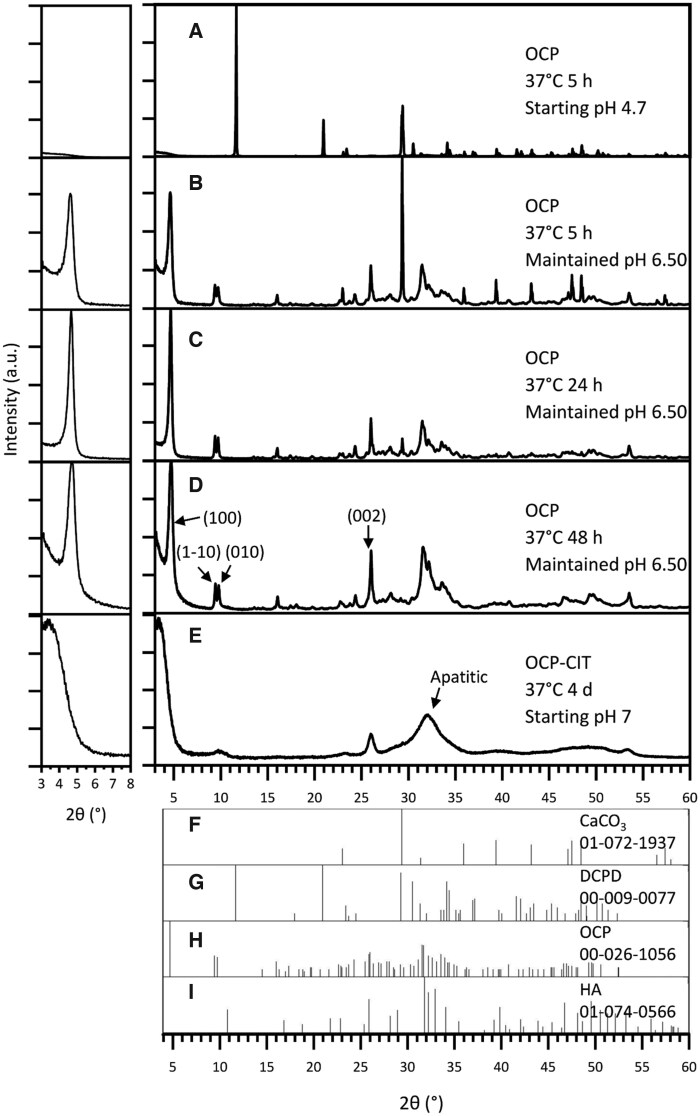
Continued.

**Table 2:** The position (2*θ*) and FWHM of the OCP (1 0 0) peak and its associated lattice parameter for OCP via the CaCO3 route

Sample	Method	Time (h)	pH	2*θ*	FWHM	*a* (Å)
OCP	CaCO_3_	5	M 6.5	4.64°	0.34°	19.03
OCP	CaCO_3_	24	M 6.5	4.67°	0.26°	18.93

The pH was maintained at a specific value (*M*).

The CaCO_3_ synthesis method was repeated in the presence of citrate with the aim of producing OCP-CIT, at 37°C for 1, 2 and 4 days, all with non-controlled pH. Before 4 days, very little, if any, precipitate was collected in the filter paper, suggesting the particles were too small. The XRD pattern of the samples collected on day 4 in Figure 2E shows broad peaks, indicating the formation of nanocrystalline HA. OCP and HA have similar crystal structures, resulting in overlapping reflections. However, the (1 −1 0) and (0 1 0) peaks align with the OCP XRD pattern, suggesting a minor OCP contribution.

#### α-TCP route

**Figure 3:**
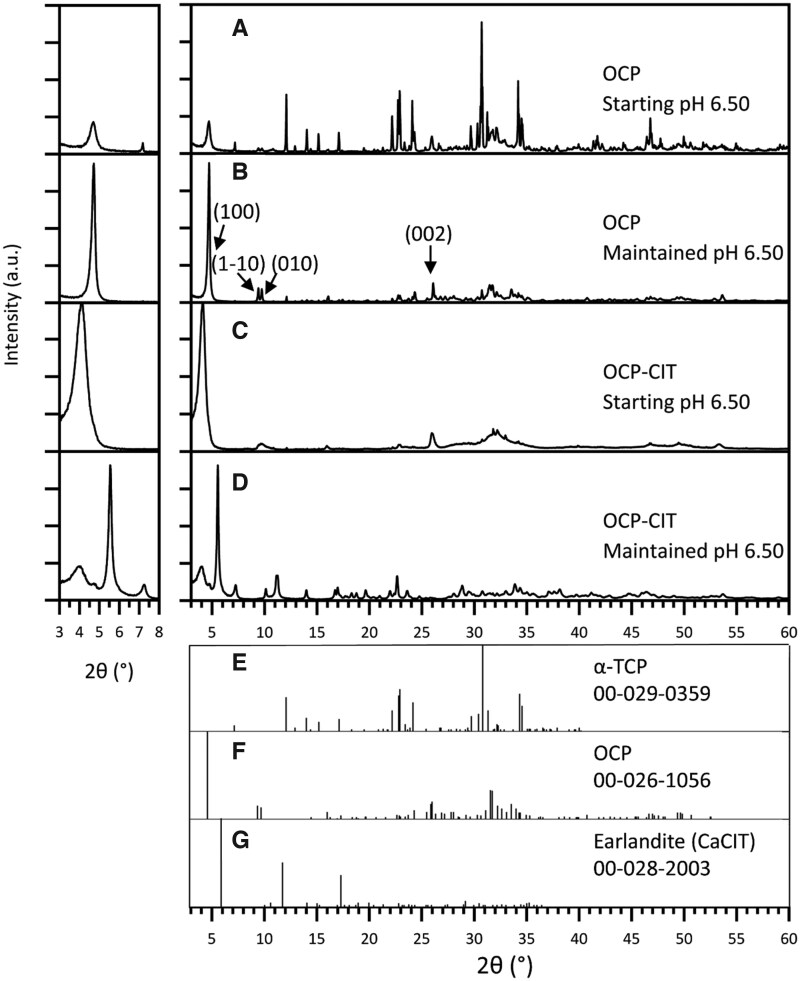
The XRD patterns of products from an OCP and OCP-CIT (α-TCP) synthesis where the pH was either started at 6.50 or maintained at 6.50. In OCP syntheses, compared to the synthesis with a starting pH of 6.50 (**A**), the amount of unreacted α-TCP was greatly reduced when the pH was maintained at 6.50 (**B**). In OCP-CIT synthesis, the synthesis with a starting pH of 6.50 (**C**) produced OCP-CIT, whereas maintaining the pH at 6.50 (**D**) resulted in CaCIT contamination. In the non-controlled reactions, the final pH for OCP and OCP-CIT was 7.4 and 7–7.4, respectively. Due to OCP’s characteristic large (1 0 0) peak around 2*θ* = 4°, the pattern is shown on a magnified scale in the inset (left) for clarity. Reference patterns for α-TCP, OCP and CaCIT are shown in (**E**–**G**), respectively.

The hydrolysis of α-TCP to produce OCP and OCP-CIT was conducted under two pH conditions: a starting pH of 6.50 that was not controlled and a maintained pH of 6.50. The XRD patterns are shown in Figure 3. In the synthesis of OCP, this resulted in samples containing 35% OCP where the starting pH was 6.50 (Figure 3A) which increased to 89% when maintaining the pH at 6.50 throughout the synthesis (Figure 3B). The (1 0 0) reflection was also narrower when using a controlled pH, indicating greater crystallinity (Table 3).

**Table 3:** The position (2*θ*) and FWHM of the OCP (1 0 0) peak and its associated lattice parameter for OCP and OCP-CIT produced the α-TCP route

Sample	Method	Time (h)	pH	2*θ*	FWHM	*a* (Å)
OCP	α-TCP	24	S 6.5	4.67°	0.32°	18.92
OCP	α-TCP	24	M 6.5	4.69°	0.18°	18.81
OCP-CIT	α-TCP	48	S 6.5	4.05°	0.74°	21.79

The pH was either starting at a specific value (*S*) or maintained at a specific value (*M*).

However, during the synthesis of OCP-CIT, using a starting pH of 6.50 produced an OCP-like phase in which the shifted reflection positions were consistent with citrate incorporation into the mineral structure (Figure 3C), whereas maintaining the pH led to contamination with calcium citrate (Figure 3D). The (1 0 0) peak shifted to a lower 2*θ* value than OCP, indicating an expanded lattice parameter, due to citrate incorporation within the crystal structure (Table 3). The broadening of the (1 0 0), (1 −1 0) and (0 1 0) peaks demonstrated a greater disorder in the OCP-CIT crystal structure compared to OCP. This disorder, and the significant difference compared to OCP, meant that the normal use of Rietveld analysis for these XRD patterns was not possible. The analysis required a well-defined phase including the locations of atoms within crystal structure. However, the presence of OCP peaks and lack of α-TCP peaks are consistent with OCP formation, although minor contributions from other phases, such as HA, cannot be entirely ruled out.

#### NMR assessment of synthesized minerals

**Figure 4:**
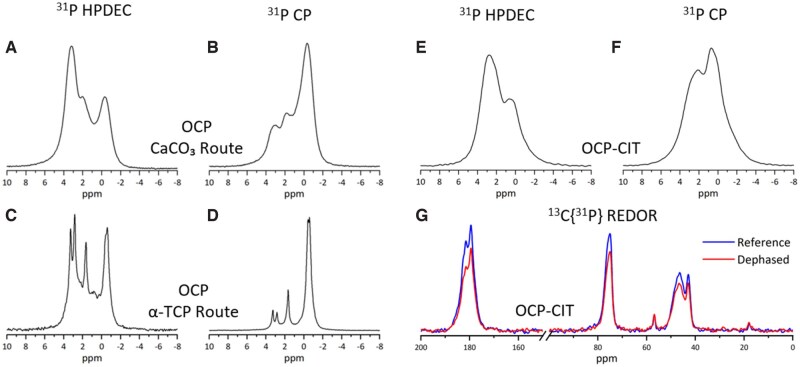
NMR evaluation of synthesized OCP and OCP-CIT minerals. The ^31^P HPDEC (left) and ^31^P CP (right, 2 ms contact time) spectra of: (**A, B**) OCP (CaCO^3^ route) 37°C 24 h; (**C, D**) OCP (α-TCP route) with pH maintained at 6.50; (**E, F**) OCP-CIT (α-TCP route) with a starting pH of 6.50 and (**G**) the ^13^C{^31^P} REDOR experiment (reference = blue, dephased = red) of OCP-CIT (α-TCP route, dephasing time = 2.9 ms). the α-TCP produced OCP minerals with significantly higher crystallinity.

The OCP produced via the CaCO_3_ and α-TCP routes was compared using NMR experiments to assess their phosphorus environments. The ^31^P HPDEC experiment magnetizes the ^31^P environments whilst high-power ^1^H decoupling removes the ^1^H–^31^P relaxation pathway. Hence, the signals in this experiment are directly proportional to the number of phosphorus atoms in each environment. The ^31^P CP experiment increases the signal from ^31^P by transferring the magnetization from ^1^H through their dipolar coupling over a range of contact times, with shorter contact times increasing the signal of ^31^P close to ^1^H. As shown by the ^31^P HPDEC and CP in Figure 4A–D, the α-TCP route produced OCP with significantly higher crystallinity than the CaCO_3_ route, as evidenced by the reduced peak broadness and greater signal resolution. Figure 4C displays some minor peaks not present in Figure 4D, which were due to a trace quantity of unreacted α-TCP. Therefore, the α-TCP route was selected for OCP-CIT synthesis and collagen mineralization.

NMR also confirmed and established the impact of citrate incorporation within the OCP structure. The ^31^P HPDEC and CP spectra of OCP-CIT (Figure 4E and F) show broader ^31^P environments and fewer resolved peaks than those of OCP, indicating greater structural disorder and loss of lattice symmetry due to citrate incorporation. ^13^C{^31^P} REDOR experiments also confirmed citrate incorporation within the OCP structure (Figure 4G). The experimental results indicate ^13^C–^31^P dipolar coupling, which is inversely proportional to the internuclear distance. The dipolar coupling reduces the intensities of carbon atoms in close proximity to phosphorus atoms. The experiment includes two parts: the reference, where the dipolar coupling is not reintroduced, and the dephased, where the dipolar coupling is reintroduced, allowing dephasing via this mechanism. Therefore, reduced signals in the dephased spectra indicate ^13^C environments close to ^31^P. The signals from OCP-CIT showed a reduced dephased signal, confirming their incorporation within the mineral structure.

### Optimizing mineralized collagen suspension for EPD

The zeta potential and conductivity of the mineralized collagen suspension were first established and modified to achieve the properties required for EPD to produce mineralized collagen films.

#### Effect of dialysis

**Figure 5:**
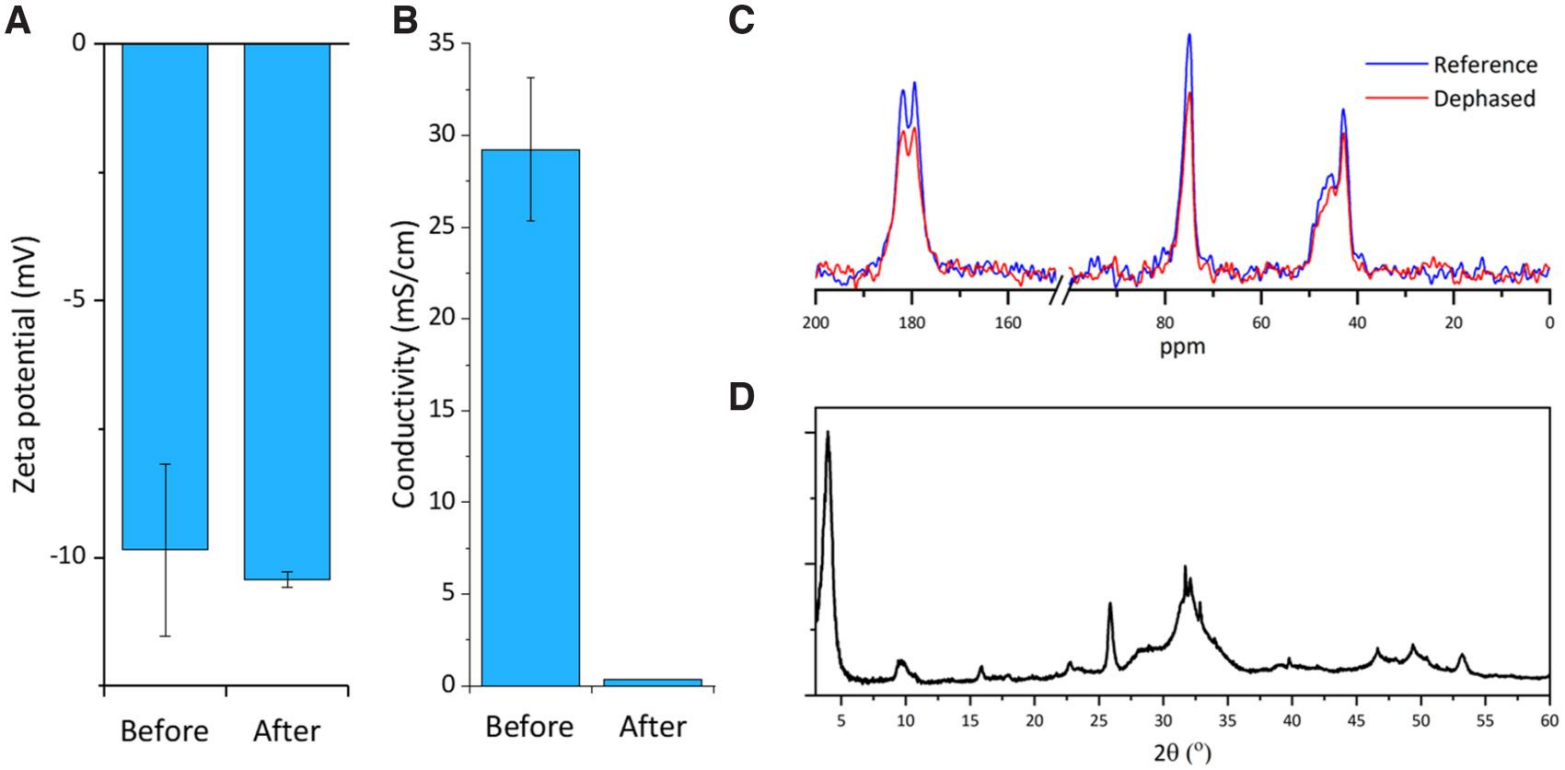
The zeta potential (**A**) and conductivity (**B**) of OCP-CIT/COLL suspension before and after dialysis against DI water (*N* = 1 measured three times). (**C**) The ^13^C{^31^P} REDOR of OCP-CIT before and after dialysis against DI water with reference = blue and dephased = red. (**D**) The XRD pattern of OCP-CIT after dialysis.

Immediately after mineralizing collagen suspension with OCP-CIT (OCP-CIT/COLL), the suspension was unstable with a tendency to flocculate, due to low zeta potential and high conductivity. During EPD, these properties would result in inhomogeneous films, a slow deposition rate and high electrolysis rates at the electrodes, leading to bubble damage to the films. Dialysing the OCP-CIT/COLL suspension against DI water significantly reduced the conductivity of the suspension, whilst the zeta potential remained relatively unchanged, as shown in Figure 5A and B. Despite the strong diffusion gradient applied during dialysis, the citrate remained incorporated within the mineral, as evidenced by the lower signal intensity in dephased spectra compared to the reference spectra in the REDOR experiment shown in Figure 5C. Additionally, the crystal structure retained the OCP-like phase following dialysis, as shown in the XRD pattern in Figure 5D compared to Figure 3C. However, the position of the (1 0 0) peak shifted to a higher 2*θ* value after dialysis, associated with a smaller lattice parameter (22.43 Å before dialysis and 21.88 Å after dialysis) likely due to the loss of citrate molecules from the OCP unit cell.

#### Effect of HyAc

**Figure 6:**
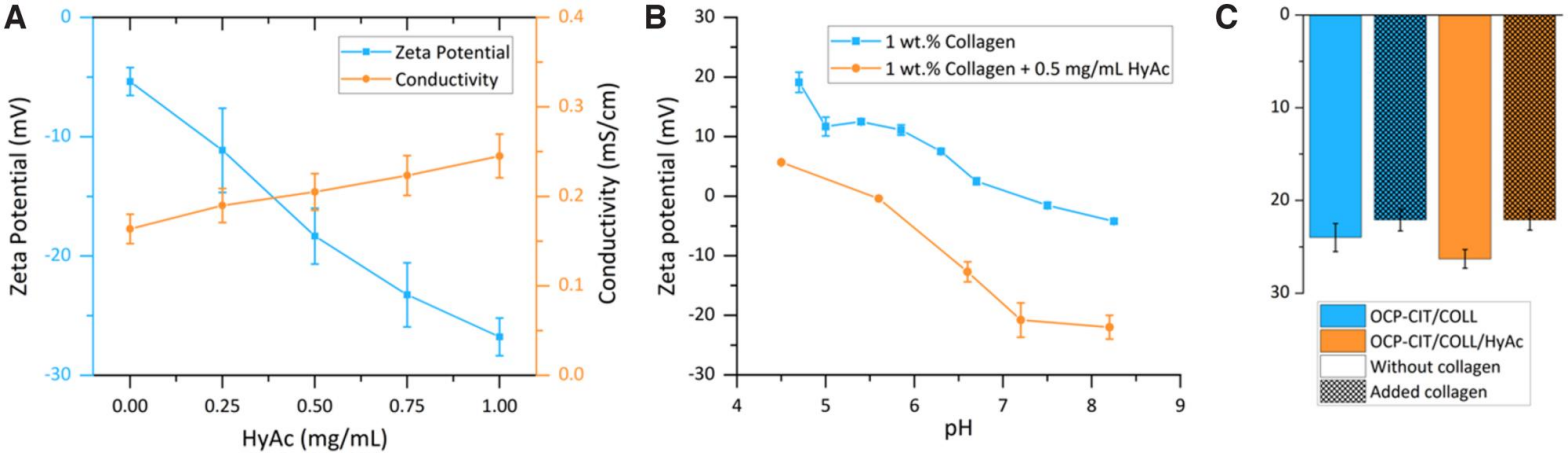
HyAc can stabilize mineralized collagen suspension. (**A**) The effect of HyAc as a processing agent on the zeta potential (blue) and conductivity (orange) of OCP-CIT/COLL suspension. (**B**) The zeta potential of collagen slurry (blue) and collagen slurry with HyAc (0.5 mg/mL, orange) over a range of pH. (**C**) The zeta potential of OCP-CIT/COLL (blue) and OCP-CIT/COLL/HyAc (orange) without (unshaded) and with (shaded) additional collagen slurry (*N* = 2 measured three times each).

The addition of HyAc significantly reduced the zeta potential of OCP-CIT/COLL suspension with only small increases in conductivity, as shown in Figure 6A. A concentration of 0.5 mg/mL HyAc was found to significantly decrease the zeta potential of collagen suspensions across a range of pH values (Figure 6B). Accordingly, the presence of HyAc resulted in a collagen–HyAc suspension with a more negative overall charge during the mineralization process. The addition of HyAc (0.5 mg/mL) either before or after mineralization did not significantly alter the resultant zeta potential nor did the addition of collagen slurry to the mineralized collagen suspensions which improved the stability of the suspension (Figure 6C).

### Evaluation of bone model

**Figure 7:**
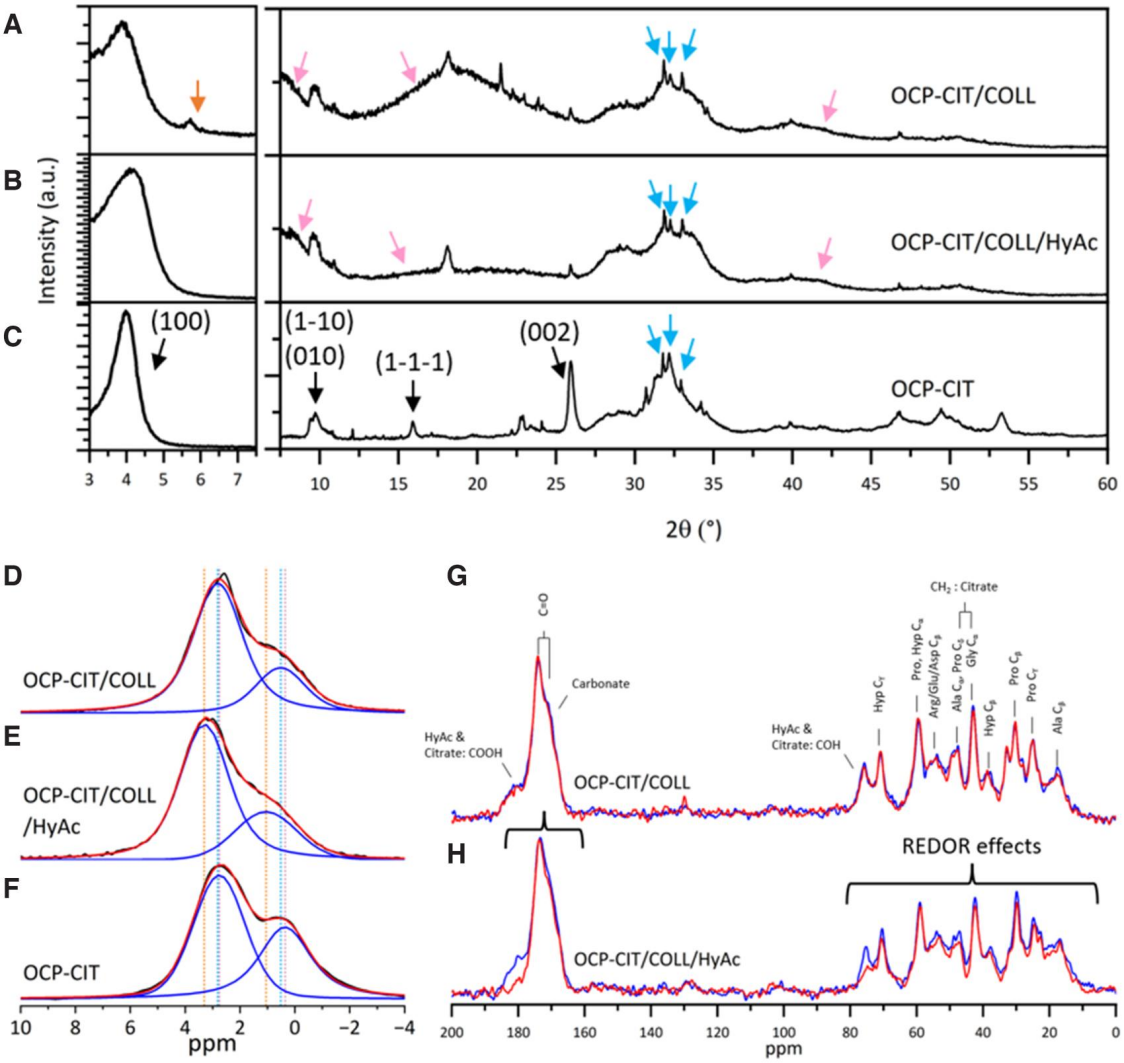
Mineralized collagen films contain bone-like collagen–mineral interfaces. XRD patterns of OCP-CIT/COLL film (**A**), OCP-CIT/COLL/HyAc film (**B**) and OCP-CIT mineral (**C**), where the reflections for OCP-CIT, HA, CaCIT and collagen were indicated by black, blue, orange and pink arrows, respectively. Due to OCP’s characteristic large (1 0 0) peak around 2*θ* = 4°, the pattern was split for clarity. ^31^P HPDEC spectra of OCP-CIT/COLL film (**D**), OCP-CIT/COLL/HyAc film (**E**) and OCP-CIT mineral (**F**), illustrating deconvolution into two fitted peaks (blue) and the overall fit (red). ^13^C{^31^P} REDOR of OCP-CIT/COLL film (**G**) and OCP-CIT/COLL/HyAc film (**H**), with the reference spectra in blue and dephased spectra in red. The regions showing REDOR effects are noted in (**H**).

The composition and collagen–mineral interface in these films were analysed using XRD and NMR techniques to establish the impact of HyAc during collagen mineralization and the success of bone model production. The XRD patterns in Figure 7A–C reveal some differences between the peaks in OCP-CIT mineral and the mineralized collagen films: slight shift in (1 0 0) reflections, broader (1 0 0) reflections in mineralized collagen films and reduced intensity of peaks at higher 2*θ* values. The (1 0 0) reflections were 3.91°, 4.10° and 4.05° for OCP-CIT/COLL film, OCP-CIT/COLL/HyAc film and OCP-CIT mineral, corresponding to lattice parameters of 22.6, 21.5 and 21.8 Å, respectively. The broad peaks in the films’ patterns were due to collagen. The reduced intensity is explained by the fixed knife edge used during XRD characterization of the mineralized collagen films, which reduced sample illumination at higher 2*θ* values. Generally, the XRD patterns showed no significant difference in crystal structure when HyAc was incorporated at the collagen–mineral interface. There were some minor phases also present in the mineralized collagen films: three peaks of HA and CaCIT contamination in the OCP-CIT/COLL films.

The mineral component of the films was also analysed using ^31^P NMR experiments, which revealed information on phosphate environments. The ^31^P HPDEC of OCP-CIT/COLL and OCP-CIT/COLL/HyAc shown in Figure 7D and E were similar: a peak containing an upfield shoulder. In comparison, the OCP-CIT spectrum contained two peaks in its ^31^P HPDEC: a dominant peak attributed to apatitic orthophosphates and a smaller, upfield peak assigned to hydrated orthophosphates and hydrogen phosphates. Deconvolutions of the spectra revealed that in the OCP-CIT mineral, ∼58% of ^31^P environments were apatitic orthophosphates, which increased to ∼74% in mineralized collagen films. Therefore, the reduced upfield peak in the mineralized collagen film spectra is consistent with a greater proportion of apatitic orthophosphates and may indicate the presence of minor HA-like environments.

The collagen–mineral interface, crucial to the properties of bone, is as important as the components themselves. The impact of HyAc on the collagen–mineral interface was evaluated using ^13^C{^31^P} REDOR experiments, as shown in Figure 7G and H. The strongest peaks are from glycine, proline and hydroxyproline amino acids, which comprise the majority of collagen residues. The OCP-CIT/COLL film did not show significant REDOR effects in regions associated with collagen or even between HyAc or citrate and mineral. In contrast, the OCP-CIT/COLL/HyAc film showed strong REDOR effects across all chemical environments, including citrate, HyAc, carbonate, as well as all the amino acids related to collagen, indicating a close arrangement between mineral and collagen. This reflects the role of HyAc in promoting mineralization in very close proximity to collagen.

Scanning electron microscopy (SEM) imaging of the OCP-CIT/COLL/HyAc films (Supplementary Figure S1) revealed regions consistent with collagen-associated mineral alongside areas of extrafibrillar mineral on the film surface. While these images support the presence of mineralized collagen fibrillar features, higher-resolution imaging would be required to definitively confirm intrafibrillar mineralization.

## Discussion

### Synthesis of OCP and OCP-CIT minerals

The successful synthesis of phase-pure OCP and OCP-CIT under physiological conditions (37°C) highlights the critical role of pH control and reaction kinetics in directing mineral phase formation. A summary of all mineralization conditions, phase assignments and characterization outcomes is provided in Table 4. For the CaCO_3_ route, maintaining a pH of 6.50 and extending the synthesis time to 24 h were essential to avoid DCPD formation and ensure complete hydrolysis. These observations align with Monma’s findings, where alkaline pH and low temperatures favour OCP over DCPD [22]. However, attempts to synthesize OCP-CIT via this route exhibited broad XRD peaks, raising ambiguity in phase assignment. While the (1 −1 0) and (0 1 0) reflections aligned with OCP, the expanded (1 0 0) lattice parameter (∼26 Å vs. literature values of 20.5–21.99 Å [20, 23]) and peak broadening could reflect either citrate-induced disorder or, more likely, nanocrystalline HA formation [32]. Citrate’s strong chelation with calcium ions likely slowed reaction kinetics, stabilizing smaller crystallites and broadening XRD patterns [33, 34]. This aligns with Davies *et al.*, who noted reduced crystallinity in OCP-CIT due to citrate incorporation [4, 20].

**Table 4:** Summary of mineralization conditions, phase assignments and characterization results for all samples^a^

Mineralization conditions						Characterization results	
Method	COLL	CIT	HyAc	Time (h)	pH	Phase assignment	^13^C{^31^P} REDOR effects
CaCO_3_				5	S 4.7	DCPD	
CaCO_3_				5	M 6.5	OCP/minor CaCO_3_	
CaCO_3_				24	M 6.5	OCP/minor CaCO_3_	
CaCO_3_				48	M 6.5	OCP/minor HA	
CaCO_3_		Y		96	S 7.0	Nano-HA/minor OCP	
α-TCP				24	M 6.5	α-TCP/minor OCP	
α-TCP				24	S 6.5	OCP/minor α-TCP	
α-TCP		Y		48	M 6.5	CaCIT	
α-TCP		Y		48	S 6.5	OCP-CIT^b^	Citrate–mineral
α-TCP	Y	Y		96	S 6.5	OCP-CIT/minor HA-like^b^	Minimal effects
α-TCP	Y	Y	Y	96	S 6.5	OCP-CIT/minor HA-like^b^	Citrate–mineral & collagen–mineral

a Phase identification was based on XRD and 1D ³^1^P solid-state NMR analyses.

b OCP-CIT refers to the OCP-like phase in which the shifted reflection positions were consistent with citrate incorporation into the mineral structure.

In contrast, the α-TCP route produced higher-purity OCP (89% phase purity) when pH was maintained at 6.50, likely due to enhanced α-TCP dissolution and calcium/phosphate ion availability [35]. However, maintaining pH during OCP-CIT synthesis led to CaCIT contamination, whereas starting at pH 6.50 without control yielded an OCP-like phase in which the shifted reflection positions were consistent with citrate incorporation into the mineral structure. This discrepancy arises from the interplay between citrate speciation and pH: higher pH promotes citrate deprotonation, increasing calcium citrate complexation [36]. The non-controlled synthesis’s final pH (7–7.4) likely favoured OCP-CIT nucleation over CaCIT, as thermodynamic driving forces for OCP increase with alkalinity [37]. NMR confirmed citrate incorporation via reduced ^31^P peak resolution in OCP-like phase and ^13^C{^31^P} REDOR dephasing, consistent with the literature [20]. Notably, the α-TCP route’s superior crystallinity (narrower XRD peaks and resolved NMR signals) and high purity were considered to be more suitable for collagen mineralization with the intended purpose of producing a bone model.

### Optimizing mineralized collagen suspensions for EPD

To enable EPD of dense mineralized collagen films, suspension conductivity and zeta potential required careful modulation. Initial mineralized collagen suspensions exhibited high conductivity (∼30 mS/cm) due to residual ions, which dialysis effectively reduced to ∼0.01 mS/cm without citrate leaching, as confirmed by retained REDOR signals. However, the suspension zeta potential remained near collagen’s isoelectric point (pH 7–7.4), leading to suspension instability. The addition of HyAc proved pivotal: it lowered the zeta potential of the collagen suspension (from −5 mV to −25 mV at 0.5 mg/mL) likely through interactions with collagen via hydrogen bonding, enhancing fibril charge homogeneity [30]. Crucially, neither HyAc nor the dialysis procedure altered phase assignment but improved EPD efficiency by mitigating against electrolysis-induced film damage, enabling uniform film deposition.

### Mineralized collagen films as bone analogues

#### Collagen–mineral interface and citrate’s dual role

The mineralized collagen films exhibited a structural transition from OCP-CIT to apatitic orthophosphates, as evidenced by ^31^P NMR (∼74% apatitic vs. 58% in OCP-CIT). This mirrors the Duer bone mineral model, where alternating OCP/HA layers form during maturation [4]. Collagen fibrils likely accelerated this transformation by providing nucleation sites, as observed in native bone [38]. However, citrate alone failed to establish a significant collagen–mineral interface in this work. Despite citrate’s theorized role in directing mineral deposition via electrostatic interactions, its high chelation capacity (CIT/Ca ratio ∼1.7) likely inhibited calcium ion availability, limiting intrafibrillar mineralization, consistent with studies showing mineral inhibition at CIT/Ca > 0.8 [39, 40].

Interestingly, despite XRD and NMR experiments indicating the presence of OCP-CIT, OCP-CIT/COLL did not show a significant interface between the citrate and mineral. Potentially, the citrate molecules had increased mobility, which reduced the REDOR effects [41]. Alternatively, a substantial proportion of citrate may have bound to the collagen fibrils, possibly due to its small size, enabling penetration into intrafibrillar spaces [42], and electrostatic interactions between the negatively charged citrate ions and positively charged regions on the collagen fibrils [43, 44].

#### Synergistic role of HyAc in collagen mineralization

The inclusion of HyAc with citrate during collagen mineralization proved transformative. REDOR experiments revealed strong ^13^C–^31^P coupling in OCP-CIT/COLL/HyAc films, indicating close proximity between both mineral and citrate, and mineral and collagen, consistent with intimate collagen-associated mineralization and suggestive of possible intrafibrillar mineral formation. HyAc’s anionic charge likely facilitated calcium ion binding near collagen fibrils, creating localized supersaturation and directing mineral nucleation towards collagen-associated domains [31]. This work agrees with the results from Shao *et al.*, which found that an anionic polymer was required to facilitate intrafibrillar mineralization in the presence of citrate ions [45]. The synergy between citrate and HyAc mirrors the function of non-collagenous proteins like poly-aspartic acid *in vivo*, suggesting HyAc can serve as a biomimetic alternative.

#### Implications for bone biomimetics

The successful integration of OCP-CIT and collagen via EPD addresses a critical gap in bone analogue development. Conventional HA-based models lack the hydrated, citrate-rich surface layer essential for bone’s mechanical resilience. By replicating both the apatitic core (via OCP-CIT) and the disordered surface (via citrate and HyAc), this work advances towards a physiologically relevant bone model. Furthermore, the use of EPD under mild conditions preserves mineral hydration, a key advantage over vacuum-based methods.

## Conclusion

This study successfully demonstrates the development of biomimetic OCP-CIT–collagen films as advanced bone analogues through the integration of pH-controlled mineral synthesis and EPD. By optimizing the hydrolysis of α-TCP under physiological conditions with precise pH regulation, OCP and OCP-CIT were synthesized, replicating bone’s hydrated, carboxylate-rich mineral interface. HyAc played a pivotal role in stabilizing mineralized collagen suspensions and promoting collagen mineralization. The resultant OCP-CIT/COLL/HyAc films exhibited a collagen–mineral architecture, dominated by apatitic orthophosphates, with citrate templating mineral nucleation and HyAc synergistically facilitating mineral–collagen interactions. While the films showed features of intrafibrillar mineralization, confirmation of such a hierarchical structure requires high-resolution imaging. This approach overcomes the limitations of HA-based models by emulating both the apatitic core and disordered surface of native bone minerals, offering a physiologically relevant platform for bone grafts and disease studies. Collectively, this work bridges fundamental chemistry and biomaterials engineering, providing a scalable film-based strategy to mimic bone’s complexity at the nanoscale and advance regenerative tissue research.

## Supplementary Material

rbaf136_Supplementary_Data

## Data Availability

The data supporting this study are available on Apollo (University of Cambridge Repository) at http://doi.org/10.17863/CAM.120589.
