# Supplementary material for: Development and optimization of electrophoretically deposited octacalcium phosphate–collagen film as bone analogues
Source: Regen Biomater. 2026 Feb 6;13:rbaf136. doi: 10.1093/rb/rbaf136 (PMC12937589; doi:10.1093/rb/rbaf136)
Supplement: rbaf136_Supplementary_Data [file rbaf136_supplementary_data.docx]

**Development and optimisation of electrophoretically deposited octacalcium phosphate-collagen film as bone analogues**

Katrina J. Staunton-Mann ^1^*, Gengyao Wei ^1^, Thomas Kress ^2^, David J. Barrett ^1^, Melinda J. Duer ^2^, Ruth E. Cameron ^1^, Serena M. Best ^1^

^1^ Department of Materials Science & Metallurgy, University of Cambridge, 27 Charles Babbage Road, Cambridge CB3 0FS, UK.

^2^ Yusuf Hamied Department of Chemistry, University of Cambridge, Lensfield Road, Cambridge CB2 1EW, UK.

*Corresponding author. Email address: kjs84@cantab.ac.uk.

Supplementary Information

1. SI Materials and Methods

Surface morphology analysis of mineralised collagen films was performed on an FEI Nova NanoSEM carried out on an FEI Nova NanoSEM in secondary mode, operated at 5 kV, spot size 2.5. Prior to imaging, the films were mounted on conducting metallic stubs using carbon tape and were gold sputter-coated with argon-gassed Emitech K550 (Emitech, France) for 2 min at a current of 20 mA.

1. Surface morphology of mineralised collagen films

The surface morphology of each side of the mineralised collagen films differed, as shown in Fig S1. Side 1 of each film faced the liquid suspension and side 2 faced the gelatine membrane during EPD. Both types of mineralised collagen films revealed a rough surface on side 1 with extraneous mineral flakes ~1 μm in length and ~0.3 μm in width, similar in morphology to OCP-CIT minerals [4]. In the OCP-CIT/COLL/HyAc films, some calcified collagen fibrils were also observed, although their fine structure is only partially resolved at this magnification. Each film exhibited a smooth surface on side 2 with a few dispersed particles approximately 0.5 μm in diameter. The mineral flake structures were also found at the edges of the films.


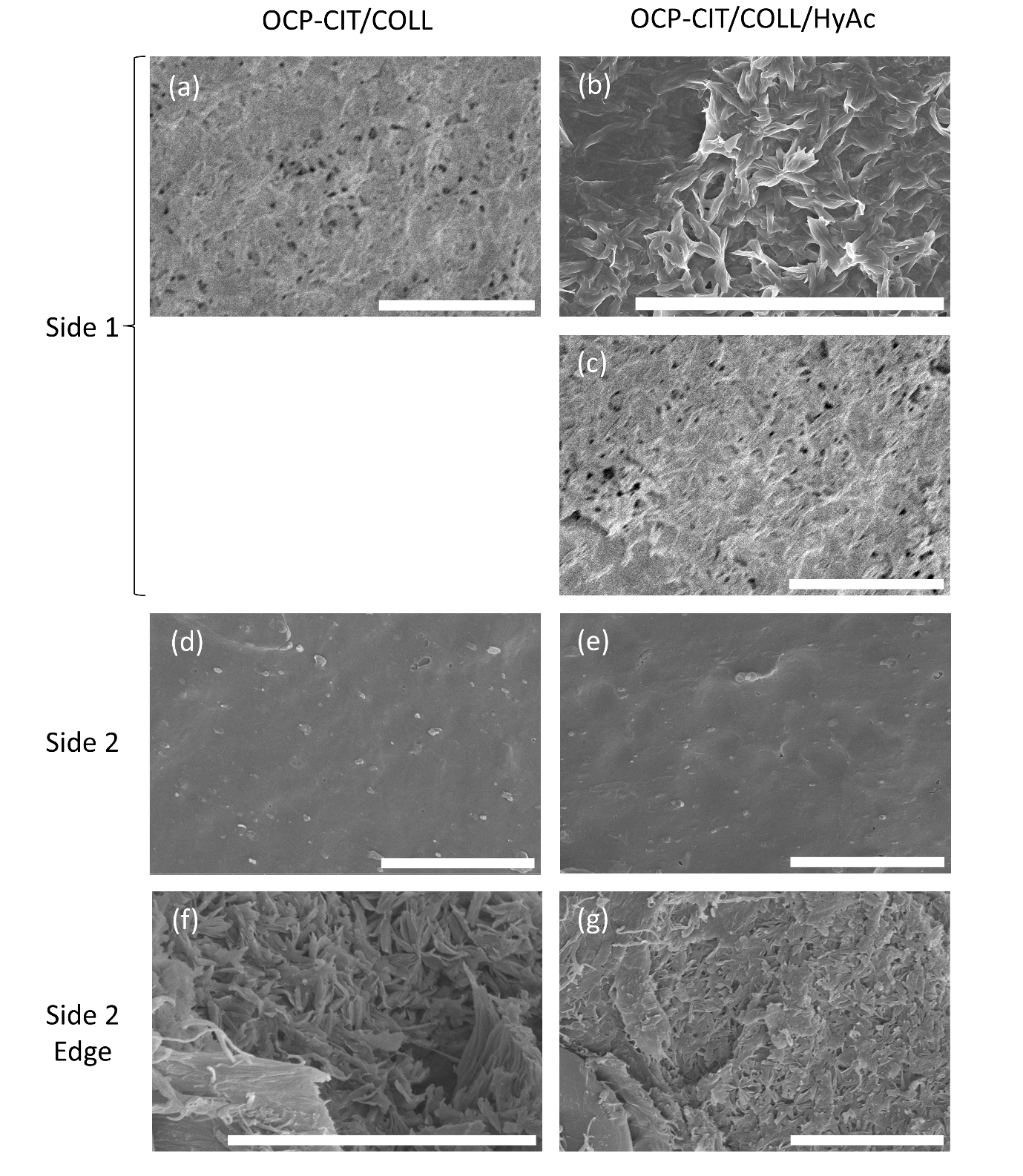


Fig. S1 SEM images of each side of OCP-CIT/COLL (a, d, f) and OCP-CIT/COLL/HyAc (b, c, e, g) films. Scale bars correspond to 10 µm.
